# Silver Nanoscale Hexagonal Column Chips for Detecting Cell-free DNA and Circulating Nucleosomes in Cancer Patients

**DOI:** 10.1038/srep10455

**Published:** 2015-05-21

**Authors:** Hiroaki Ito, Katsuyuki Hasegawa, Yuuki Hasegawa, Tadashi Nishimaki, Kazuyoshi Hosomichi, Satoshi Kimura, Motoi Ohba, Hiroshi Yao, Manabu Onimaru, Ituro Inoue, Haruhiro Inoue

**Affiliations:** 1 Digestive Disease Center, Showa University Koto Toyosu Hospital, Tokyo, Japan; 2 Mytech Inc., Kobe, Japan; 3 Unit of Organ Oriented Medicine, Division of Digestive and General Surgery, Department of Medicine, Ryukyu University, Okinawa, Japan; 4 Division of Human Genetics, National Institute of Genetics, Mishima, Japan; 5 Department of Laboratory Medicine and Central Clinical Laboratory, Showa University Northern Yokohama Hospital, Yokohama, Japan; 6 Institute of Molecular Oncology, Showa University, Tokyo, Japan; 7 Graduate School of Material Science, University of Hyogo, Hyogo, Japan

## Abstract

Blood tests, which are commonly used for cancer screening, generally have low sensitivity. Here, we developed a novel rapid and simple method to generate silver nanoscale hexagonal columns (NHCs) for use in surface-enhanced Raman scattering (SERS). We reported that the intensity of SERS spectra of clinical serum samples obtained from gastrointestinal cancer patients is was significantly higher than that of SERS spectra of clinical serum samples obtained from non-cancer patients. We estimated the combined constituents on silver NHCs by using a field emission-type scanning electron microscope, Raman microscopes, and a 3D laser scanning confocal microscope. We obtained the Raman scattering spectra of samples of physically fractured cells and clinical serum. No spectra were obtained for chemically lysed cultured cells and DNA, RNA, and protein extracted from cultured cells. We believe that our method, which uses SERS with silver NHCs to detect circulating nucleosomes bound by methylated cell-free DNA, may be successfully implemented in blood tests for cancer screening.

Early diagnosis is important for improving the chances of survival for a patient with cancer. Blood tests are widely used for diagnosis because they are easy to perform and are minimally invasive. They have been used to tumor markers^1^,^2^,^3^, cancer-related nucleic acids^4^,^5^, and circulating tumor cells (CTCs)^6^,^7^ for cancer diagnosis.

Although tumor markers have been implemented in practice, their sensitivity is generally low^8^,^9^. Hence, tumor markers must be used in combination with other tests. Real-time quantitative reverse transcription-polymerase chain reactions have also been used to show that cancer-related mRNA is associated with the prognosis of patients with esophageal cancer^10^,^11^. Moreover, CTCs indicate the prognosis of patients with gastric cancer^7^, and they are useful for diagnosis, estimation of prognosis, and determination of treatment efficacy in patients with breast^6^, prostate^12^, lung^13^, and colorectal cancers^14^. However, the sample preparation methods and tests required to analyze CTCs are more complicated than a routine clinical blood test. Additionally, the sensitivity and specificity of the technique varies^15^,^16^. Therefore, CTCs analysis is not widely used.

Cell-free nucleic acids (cfNAs), first described in 1948^17^, have been used as important biomarkers of cancer since 1994^18^,^19^,^20^,^21^. cfNAs, which include cell-free DNA (cfDNA), mRNA, and microRNA (miRNA), are present in high concentrations in the blood of cancer patients^22^,^23^,^24^,^25^,^26^,^27^. However, the analysis of mRNA^22^ and miRNA^23^,^24^ in blood is highly specific and sensitive, its usefulness is controversial^25^,^26^,^27^. The concentration of methylated cfDNA present in the blood of cancer patients is higher than that in normal individuals, and it is correlated with CTCs^28^,^29^.

A nucleosome is composed of a histone octamer core bound by a 200 base pair–long DNA strand. Although circulating nucleosomes that originate from apoptotic cells are detected in the blood of patients with benign diseases as well as patients with cancer^30^, their serum level increases over the course of cancer progression^31^ and tumor cell apoptosis owing to anticancer therapy^32^,^33^. Moreover, cfDNA methylation and histone modification of circulating nucleosomes are frequently observed in the blood of cancer patients^34^. Although histones are positively charged, acetylation of the histone tail decreases the positive charge. However, methylation of the binding DNA is strongly related to the methylation of the histone tail of the nucleosome^35^. Because histone demethylation is a rare event^36^, the methylated histone remains positively charged. This suggests that there are more circulating methylated nucleosomes in the blood of cancer patients than in the blood of patients with benign diseases or in the blood of healthy individuals; it also suggests that circulating nucleosomes with positively charged histones have the potential to be diagnostic and monitoring markers for cancer.

Surface-enhanced Raman scattering (SERS) using a laser beam is widely used in industrial microanalysis, and has been employed in biological research^37^. Recently, SERS has also been used for cancer diagnosis^38^. Although blood analysis using SERS is advantageous in that very small amounts of constituents can be detected, including CTCs^38^, nucleic acid^39^, ribonucleic acid^40^, proteins^41^, and lipids^42^, preparing nanoscale hexagonal columns (NHCs) for use in SERS and conducting measurements is time-consuming.

To address this problem, we developed a novel rapid and simple method to generate silver NHCs for use in SERS on the surface of a chip made of phosphor bronze^43^. We then used the negatively charged silver NHC chips to obtain SERS spectra generated by circulating nucleosomes with positively charged histone in patients with gastric cancer and those with colorectal cancer. We then compared these spectra with those obtained using individuals with benign diseases. We showed that the intensity of Raman scattering spectra of clinical serum samples obtained from cancer patients was significantly higher than those of clinical serum samples obtained from patients with benign diseases^43^. In contrast, no relationship was observed between the intensity of the Raman scattering spectrum and the concentration of total protein or albumin in the serum sample. Although this indicated that silver NHCs could be used in a simple and sensitive blood test for cancer diagnosis, the combined constituents on the surface of the NHC chips could not be identified. We aimed to collect these constituents by liquation or by scraping before laser irradiation, although the majority of constituents were collected from single-strand DNA. This suggests that our collection process was inadequate because clinical serum samples contained little single-strand DNA.

In this study, we estimated the combined constituents on the surface of the silver NHC chip by using a field emission–type scanning electron microscope (SEM), Raman microscopes, and a 3D laser scanning confocal microscope. Although we observed microscopically visible nodules on the silver NHC chip in samples of lysed cultured cells, extracted protein, fractured cultured cells, and clinical serum, Raman scattering spectra were observed only in samples of physically fractured cells and clinical serum. DNA and RNA extracted from cultured cells did not produce any nodules on the silver NHC chip.

In conclusion, we suggest that our new simple and rapid method, which uses SERS with silver NHCs to detect circulating nucleosomes bound by methylated cell-free DNA, may be successfully implemented in blood tests for cancer screening.

## Results

### Transition of the structure on the surface of silver NHC chips

**Figure 1:**
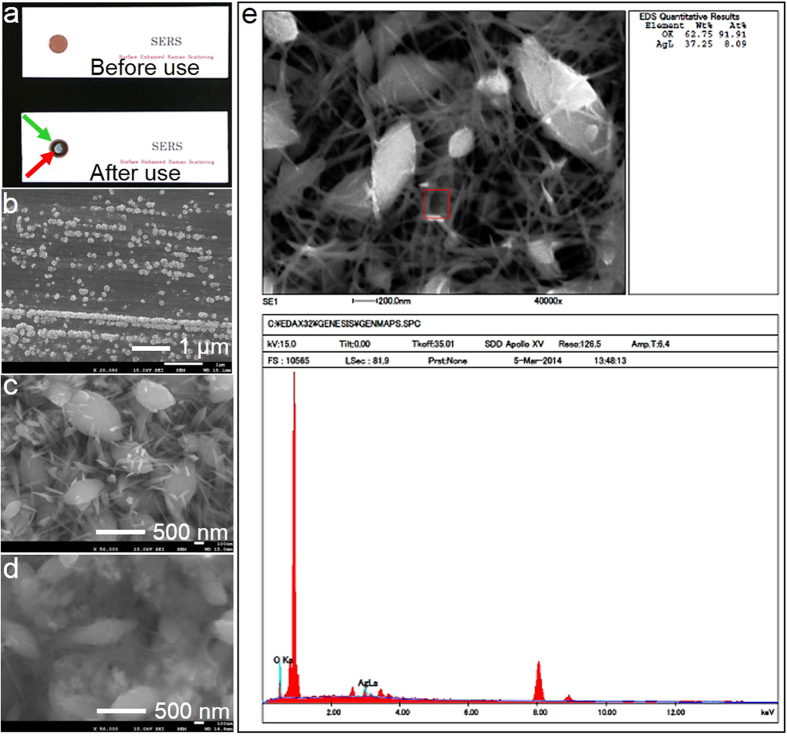
Morphological transition and composition analysis of the sturucture on the surface of a silver NHC chip. **a,** The surface of Proteo®chip, a biochip with silver NHCs on a phosphor bronze plate with a round shape, was recorded by using an SEM (JSM-7001F, JEOL Ltd., Tokyo). The color of the area where sodium hypochlorite was added changed from bronze to black (green arrow) from bronze, and then to white (red arrow) after laser irradiation. **b,** Silver NHCs were observed on the surface of the chip. **c,** After adding sodium hypochlorite onto the chip, structures composed of egg-shaped masses and thorns were observed on the chip surface. **d,** Fluff- and bridge-like components were also observed after adding a clinical serum sample obtained from a patient with Stage IIIa gastric cancer. **e,** Composition analysis of the surface of a silver NHC chip after adding sodium hypochlorite by using energy dispersive X-ray spectroscopy. The atomic percent of oxygen (91.91%) suggested that the chip surface mainly comprised silver superoxide under oxygen-rich conditions. Chlorite was hardly observed.

Figure 1a shows the Proteo®chip before and after use. The chip surface was recorded using an SEM (JSM-7001F, JEOL Ltd., Tokyo). The color of the area where sodium hypochlorite was added changed from bronze to black (green arrow), and then to white (red arrow) after laser irradiation. Silver NHCs were observed on the chip surface (Fig. 1b). After adding sodium hypochlorite onto the chip, structures composed of egg-shaped masses and thorns were observed (Fig. 1c). Fluff- and bridge-like components were also observed after adding a clinical serum sample obtained from a patient with gastric cancer (Fig. 1d).

The atomic percent of oxygen was 91.91%, and chlorine atoms were hardly detected (Fig. 1e). This suggested that the chip surface mainly comprised silver superoxide (Ag_2_O_3_) under oxygen-rich conditions.

### Structures on the surface of silver NHC chips after adding clinical serum samples

**Figure 2:**
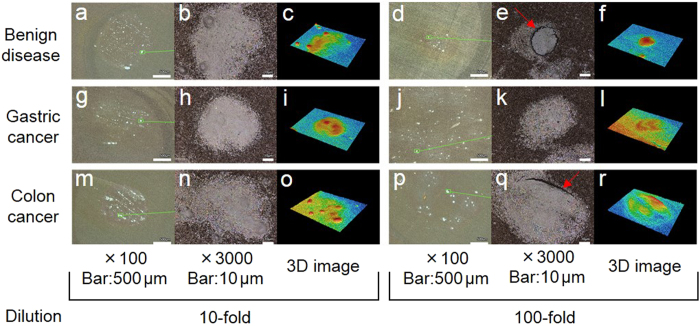
Shape and color histograms of the structure on the surface of silver NHC chips with clinical serum samples by using a 3D laser scanning confocal microscope. These are typical images. Whitish nodules were observed on the chip surface in all serum samples obtained from patients with benign diseases (gallstone, **a**, **d**), gastric cancer (Stage IIIa, **g**, **j**), and colorectal cancer (Stage IV**, m**, **p**). In serum samples diluted 10-fold, there was no clear difference among the chip surfaces after the addition of samples obtained from patients with benign diseases (**a**), gastric cancer (**g**), and colorectal cancer (**m**). In the sample obtained from a patient with a benign disease, the nodules on the chip surface with the addition of the serum sample diluted 100-fold were fewer and smaller than those with the addition of the sample diluted 10-fold (**d**). In samples obtained from patients with malignancies, nodules were observed in relatively large areas; there were no remarkable differences in the size and number of nodules between the samples diluted 10-fold (**g**, **m**) and 100-fold (**j**, **p**). There was no notable difference between the nodules in samples obtained from patients with gastric cancer and those with colorectal cancer. The number of nodules more than 10 μm in diameter in the samples diluted 10- and 100-fold was 51 and 19, 93 and 90, and 78 and 56 in the serum samples obtained from the patients with benign diseases, gastric cancer, and colorectal cancer. In magnified view (**b**, **e**, **h**, **k**, **n**, **q**), nodules with the addition of samples diluted 100-fold had clear cracks (red arrows in **e**, **q**). Histograms of the crystal of clinical serum samples on the chip were recorded by using a 3D laser scanning confocal microscope.

Whitish nodules were observed on the surface of the silver NHC chip in all serum samples obtained from patients with benign diseases (gallstone, Figs. 2a,2d), gastric cancer (Stage IIIa, Figs. 2g,2j), and colon cancer (Stage IV, Figs. 2m,2p). In serum samples diluted 10-fold, no clear difference was observed among the surfaces of the silver NHC chip with the addition of the samples obtained from patients with benign diseases (Fig. 2a), gastric cancer (Fig. 2g), and colorectal cancer (Fig. 2m). In the sample obtained from patients with benign diseases, fewer and smaller nodules were observed on the surface of the silver NHC chip with the addition of the serum sample diluted 100-fold, as compared to the number and size of nodules obtained with the addition of the sample diluted 10-fold (Fig. 2d). In samples obtained from patients with malignancies, nodules were observed in a relatively large area, and no notable differences in terms of the size and number of nodules were observed between the samples diluted 10-fold (Figs. 2g,2m) and those diluted 100-fold (Figs. 2j,2p). There was no significant difference between the crystals in the samples obtained from patients with gastric cancer and those obtained from patients with colorectal cancer. The number of nodules more than 10 μm in diameter in the samples diluted 10- and 100-fold was 51 and 19, 93 and 90, and 78 and 56 in the serum samples obtained from the patients with benign diseases, gastric cancer, and colorectal cancer. In a magnified view, a crack in the crystal was observed in samples diluted 100-fold (Figs. 2e,2q, red arrows). The whitish nodules in ×100 view were observed as a combination of hillocks and hills (samples diluted 10-fold: Figs. 2c,2i,2o) and hills (samples diluted 100-fold: Figs. 2f,2l,2r).

### Structures on the surface of silver NHC chips after adding samples from cultured tumor cells

For samples of chemically lysed cultured cells including Kato III, MKN45, CW-2, PK45-P, and NHDF-Neo, the ranges of concentrations of extracted DNA, RNA, and protein were as follows: double-strand DNA, 4.8−66.8 μg/ml; total RNA, 6.0−26.5 μg/ml; and protein, 2.4−4.0 mg/ml. Their final concentrations were adjusted by dilution with distilled water to 5 μg/ml for double-strand DNA, 5 μg/ml for total RNA, and 2 mg/ml for protein to match the samples of chemically lysed cultured cells.

**Figure 3:**
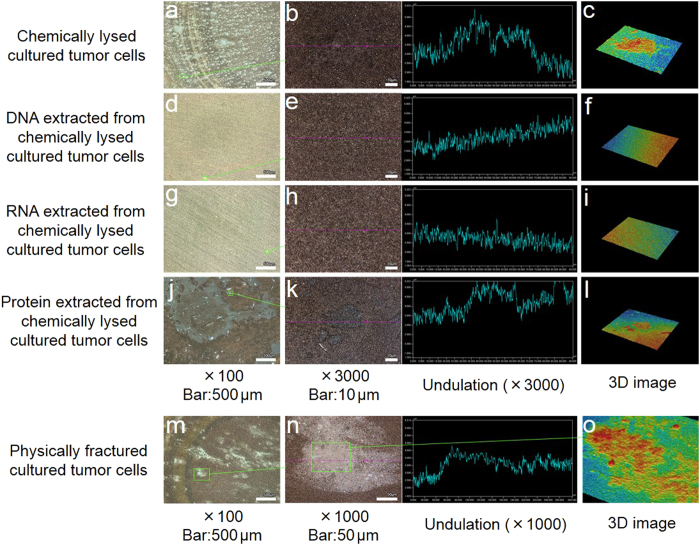
Structure of the surface of silver NHC chips with cultured cell samples by using a laser microscope. These are typical images using KATO-III. **a,** In ×100 view, many small and whitish nodules were recognized on the chip surface after adding a sample of chemically lysed cultured tumor cells. No nodules were observed after adding samples of extracted DNA (**d**) or RNA (**g**). After adding the extracted protein sample, small nodules and starch-like structures were observed (**j**). After adding a sample of physically fractured cultured tumor cells, many relatively large and whitish nodules were observed (**m**). In magnified (×3000 or ×1000) and 3D views, the largest nodule on the chip surface in ×100 view resembled a plain and hillocks (chemically lysed cultured tumor cells and extracted protein samples, **b**, **c**, **k**, **l**) or a hill (physically fractured cultured tumor cells, **n**, **o**). No on-chip structure was observed after adding samples of extracted DNA (**e**, **f**) or RNA (**h**, **i**).

In ×100 view, many small and whitish nodules were observed on the chip surface after adding samples of chemically lysed cultured tumor cells (Fig. 3a). In contrast, no obvious nodules were observed after adding extracted DNA (Fig. 3d) and RNA (Fig. 3g) samples. After adding the extracted protein sample, small nodules and starch-like structures were observed (Fig. 3j). After adding the sample of physically fractured cultured tumor cells, many relatively large and whitish nodules were observed (Fig. 3m). In ×3000 and 3D views, the largest nodule in ×100 view resembled a plain or a hillock (chemically lysed cultured tumor cells and extracted protein samples: Figs. 3b,3c,3k,3l), or coarse hills (physically fractured cultured tumor cells: Figs. 3n,3o). No notable structure was observed after adding extracted DNA and RNA samples (Figs. 3e,3f,3h,3i).

### Raman scattering spectrum of samples from cultured cells

**Figure 4:**
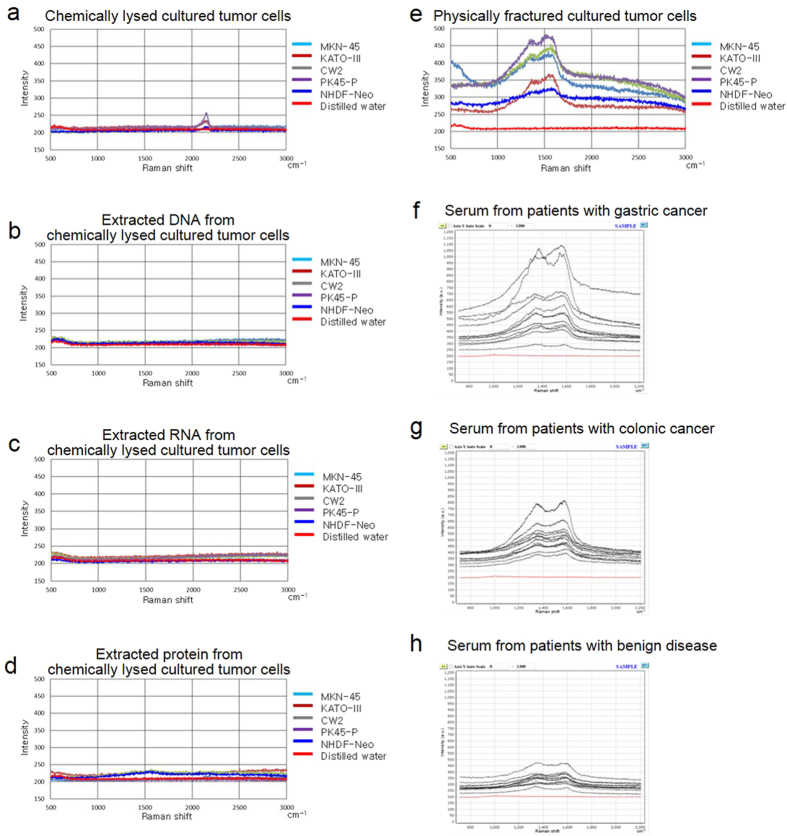
Raman scattering spectrum of cultured cell and clinical serum samples. Raman scattering spectra of chemically lysed cultured cells, (**a**); extracted DNA, (**b**); extracted RNA, (**c**); extracted protein, (**d**); physically fractured cultured cells, (**e**); and clinical serum samples obtained from patients with gastric cancer, (**f**); colorectal cancer, (**g**) ; benign diseases, (**h**) were recorded by using a Raman microscope (RAM-300, Lambda Vision, Inc.). The Raman scattering spectra for the gastric cancer (**f**), colorectal cancer (**g**), and benign diseases (**h**) groups are from our previous report^43^. No notable Raman scattering spectra were observed in samples of chemically lysed cultured cells (**a**) or those of extracted DNA (**b**), RNA (**c**), or protein (**d**). In contrast, marked Raman scattering was observed in samples of physically fractured cultured cells, and the intensity of the spectrum in a sample of normal dermal fibroblasts was relatively lower than that in samples of tumor cells (**e**). The spectra in samples of tumor cells and normal dermal fibroblasts were similar to those in clinical serum samples obtained from patients with malignancies (**f, g**) and benign diseases (**h**).

No notable peaks in Raman scattering spectra were observed for the sample of chemically lysed cultured cells, except for a weak spectral peak near 2000 cm^−1^, which indicated a non-specific organic molecule (Fig. 4a). No prominent peaks in Raman scattering spectra were observed for the samples of extracted DNA (Fig. 4b), RNA (Fig. 4c), or protein (Fig. 4d). However, significant peaks in Raman scattering spectra were observed for the samples of physically fractured cultured cells; the intensity of the spectra of samples of normal dermal fibroblasts was relatively lower than that of samples of tumor cells (Fig. 4e). Despite differences in the measurement and recording conditions, the spectra of the samples of tumor cells and normal dermal fibroblasts were similar to those of clinical serum samples obtained from patients with malignancies (gastric cancer: Fig. 4f; colorectal cancer: Fig. 4g) and benign diseases (gallstone, cholecystitis, and achalasia: Fig. 4h).

### SERS focusing on crystals on silver NHC chips

**Figure 5:**
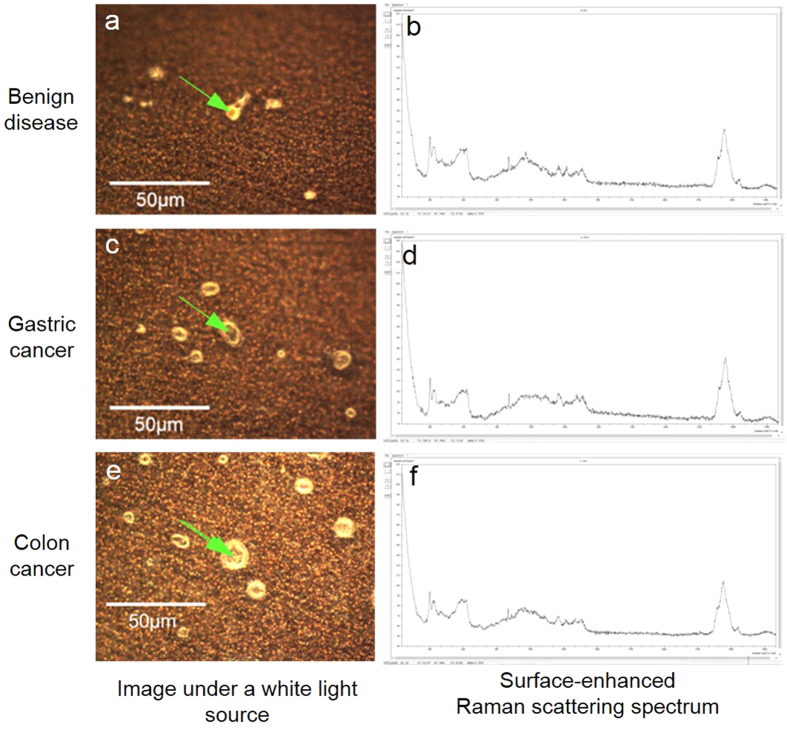
Surface-enhanced Raman scattering spectra of a crystal from serum samples on silver NHC chips. These are typical images. After applying a laser beam to the center of the crystal from clinical serum samples obtained from patients with benign diseases (gallstone, **a**), gastric cancer (Stage IIIa, **c**), and colorectal cancer (Stage IV, **e**), surface-enhanced Raman scattering spectra (**b, d, f**) were obtained.

After a laser beam was applied to the center of the crystal, SERS spectra were acquired for all samples obtained from patients with benign diseases (gallstone: Figs. 5a,5b), gastric cancer (Stage IIIa: Figs. 5c,5d), and colorectal cancer (Stage IV: Figs. 5e,5f).

RGB color histograms of the structure on the surface of silver NHC chips with clinical serum samples

**Figure 6:**
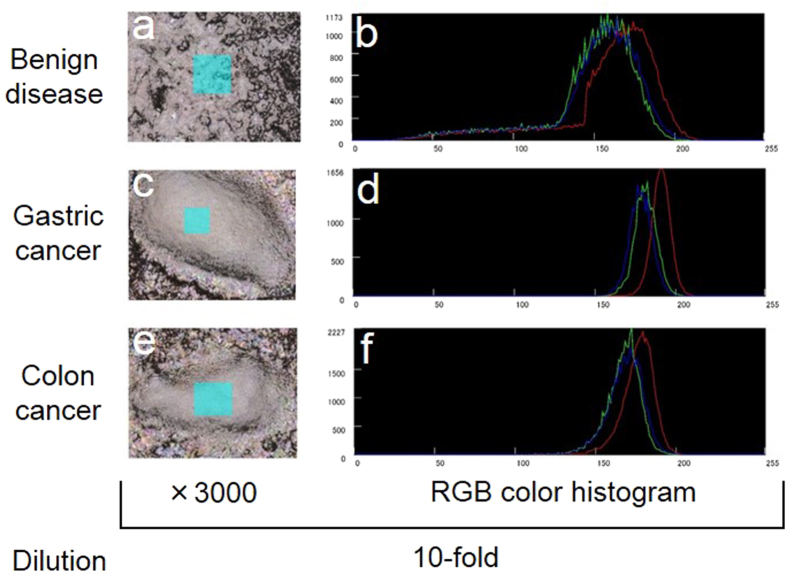
RGB color histograms of the structure on the surface of silver NHC chips with clinical serum samples obtained using a 3D laser scanning confocal microscope. These are typical images. Histograms of the crystal of clinical serum samples on the chip were recorded using a 3D laser scanning confocal microscope. The RGB color histograms of the crystals (**d**, **f**) of clinical serum samples obtained from patients with gastric cancer (Stage IIIa, **c**) and colon cancer (Stage IV, **e**) were narrower than the histogram of the crystal (**a**) of a clinical serum sample obtained from a patient with a benign disease (gallstone, **b**).

The RGB color histogram of the crystal of clinical serum samples obtained from a patient with gastric (Stage IIIa, Figs. 6c,6d) or colon cancer (Stage IV, Figs. 6e,6f) was narrower than that of the clinical serum sample obtained from patients with benign diseases (gallstone, Figs. 6a,6b).

## Discussion

Early and precise diagnosis is important to improve a cancer patient’s chance of survival. We developed a silver NHC chip to detect cancer-related constituents by using the SERS method. Although a target molecule can be selectively detected by using a specific method such as antibody-based detection, we used negatively charged silver NHCs to detect circulating nucleosomes with positively charged histones. We previously reported the utility of the silver NHC chip to diagnose gastric and colorectal cancers by using SERS analysis. Although we attempted to collect the combined constituents on the silver chip by distilled water liquation or by scratching using a cell scraper, the collected constituents mostly comprised single-strand DNA. This suggested that our collection process was incomplete because the pre-measurement clinical serum samples contained less single-strand DNA.

SERS can detect a single molecule^44^ and DNA methylation^45^. Additionally, the intensity of SERS correlates with the quantity of the material. Thus, the pattern of the SERS spectrum depends on the particular constituents and their quantities present in the sample, with different samples having diverse spectral patterns. Although the constituents of a substance in an unknown sample can be deduced by inspection of the SERS pattern and unknown constituents can be distinguished as potential biomarkers, methods such as liquid chromatography, electrophoresis, and mass spectrometry must also be used to determine the identity of such constituents. Because collecting all of the combined constituents on the silver NHC chip is technically difficult, in this study, we analyzed the transition of the chip surface after adding various samples and determined the combined constituents on the surface of the chip.

After adding sodium thiosulfate pentahydrate, the main component of the surface on the chip was suggested to be silver superoxide by composition analysis via energy dispersive X-ray spectroscopy using an SEM (Fig. 1f). Under oxygen-rich conditions, silver (III) oxide (Ag_2_O_3_) is generated (Fig. 1g). The color of the chip surface changed from bronze to black after adding sodium thiosulfate pentahydrate. This also suggested the generation of Ag_2_O_3_^46^. As the shape of the structure on the chip surface changed after adding clinical serum samples obtained from patients with cancer, the constituents in the clinical serum samples and silver oxide may mix and crystallize (Fig. 1e). Because the chip surface is negatively charged, positively charged constituents in the serum should bind to the chip surface^43^. Most positively charged constituents in the serum are presumably gamma globulins and circulating nucleosomes; gamma globulins should not exist in cultured cell samples. This suggests that the negatively charged silver NHC chip trapped circulating nucleosomes.

In clinical serum samples diluted 10-fold, no notable difference was observed between the chip structures of benign disease and cancer samples. In samples diluted 100-fold, the structures of the benign disease sample were fewer and smaller than those of the cancer samples. No difference was observed between benign disease and cancer samples diluted 10-fold, consistent with the previous work^43^, suggesting that the clinical serum sample diluted 10-fold saturates the silver NHC chip. The difference between benign disease and cancer samples diluted 100-fold suggests that the combined constituents on the chip in the sample obtained from patients with cancer are more than those in the sample obtained from patients with benign diseases. We should confirm the optimal dilution of serum samples to distinguish cancer from benign diseases by measuring many clinical samples. Cracks in the crystal in the sample diluted 100-fold may be a result of the concentration of combined components, and we presume that the cracks may be caused by the inequality of the constituent component and structure of the crystal. A difference in the RGB color histogram of the structure on the silver NHC chip was observed between benign disease and cancer (Figs. 6b,6d,6f) samples. Although it is unclear whether this difference originated from the components or shape of the structure on the chip in this study, the results suggest that such a histogram may be useful for cancer diagnosis.

**Figure 7:**
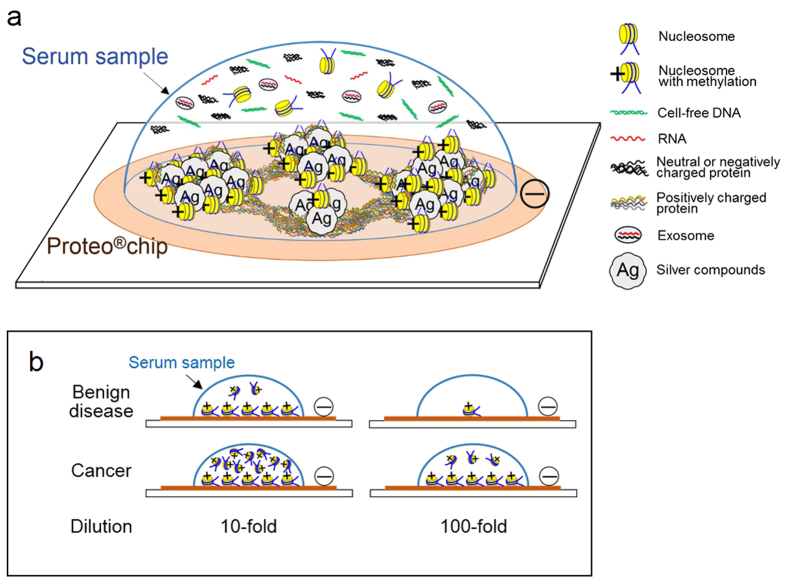
Combination of serum substances on a silver NHC chip. The surface of the Proteo®chip is negatively charged to trap positively charged histones in the circulating nucleosome. Because methylated DNA in the circulating nucleosomes cause histone methylation, the histones remain positively charged. This suggests that the circulating nucleosomes with methylation efficiently bind to the surface of the Proteo®chip (**a**). Clinical serum samples of patients with benign disease can be distinguished from those of cancer patients in diluted samples as well (**b**).

Samples from cultured cells, chemically lysed cultured cells (Fig. 3a), extracted protein (Fig. 3j), and physically fractured cultured cells (Fig. 3m) produced visible structures on the chip as inspected by using a laser microscope. The on-chip structures were different in each sample, and only the physically fractured cells showed Raman scattering spectra (Fig. 4e). Because the on-chip structure produced by samples of chemically lysed cultured cells and protein were relatively smaller and thinner than those produced by physically fractured cell samples in the 3D view, a precise spectrum might not be observed. These data indicate that chemically reduced compounds, as well as extracted DNA, RNA, and protein, show no definite Raman scattering spectrum. They suggest that incompletely reduced compounds such as nucleosomes may bind to the chip surface. Because the silver NHC chip is negatively charged, positively charged constituents should bind with the chip. Therefore, it is suggested that the positively charged histone core of the circulating nucleosomes combine with the chip. Although gamma globulins are positively charged in serum, they are absent in samples from cultured cells. The intensities of the Raman scattering spectra of cultured tumor cells were higher than those of cultured normal dermal fibroblasts (Fig. 4e). Our data are consistent with reports that methylation of DNA and histone in tumor cells is more pronounced than in normal cells^47^,^48^,^49^. Moreover, the patterns of Raman scattering in tumor cells and normal dermal fibroblasts were similar to those of serum samples obtained from patients with cancer and benign diseases, respectively (Figs. 4f,4g,4h). These data suggest that our assay detects cancer-related constituents, and hence cell-free nucleosomes with methylation (Fig. 7a). Diluted clinical serum samples of patients with benign diseases are distinguished from those of patients with cancer (Fig. 7b) because the silver NHC chip is saturated by a certain amount of circulating nucleosomes.

A Raman microscope with a sighting device observed more detailed Raman scattering spectra than one without a sighting device, by focusing on the crystal on the chip (Figs. 5a,5c,5e). Because these detailed spectra are mainly generated by an SERS phenomenon (Figs. 5b,5d,5f), the Raman microscope with a sighting device is considered useful for sample composition analysis.

There are some limitations to this study. We have not yet directly confirmed the combined constituents on the silver NHC chip. In addition, the silver NHC chip may detect unknown tumor-related biomarkers other than cell-free nucleosomes with methylation. Thus, we have sought to collect the constituents from the chip surface. Fluorescence labeling may reveal constituents that bind with silver superoxide in the crystal on the chip. Moreover, further clinical serum samples should be analyzed to confirm the usefulness of this method for cancer diagnosis. We have already collected more than 100 clinical serum samples and are currently preparing the analysis. In addition, we are developing a new Raman microscope with a sighting device focusing on the center of the crystal on the chip, to obtain detailed Raman spectra, accurately.

In conclusion, we suggest that the simple and rapid method using SERS with silver NHCs described here, by detection of circulating nucleosomes bound by methylated cell-free DNA, may be successfully implemented in blood tests for cancer screening.

## Methods

### Ethics statements

This study was performed according to the principles of the Declaration of Helsinki and was approved by the Institutional Review Board of the Showa University, Northern Yokohama Hospital (No. 1212-02) and Showa University, Koto Toyosu Hospital (No. 14T5008). This study was registered with the University Hospital Medical Information Network in Japan, number 000009818. We explained the study protocol to patients before they gave written informed consent.

### Patients

We studied patients who underwent treatment for esophageal, gastric, and colorectal cancer, or for benign diseases. The inclusion criteria were as follows: (i) presence of carcinoma histologically proven from a biopsy (for patients with esophageal, gastric, or colorectal cancer); (ii) absence of malignant disease on computed tomography (patients with benign diseases); (ii) clinical solitary tumor; (iv) no prior treatment of endoscopic resection, chemotherapy, or radiation therapy; (v) aged between 20 and 80 years; (vi) Eastern Cooperative Oncology Group performance status^50^ of 0 or 1; (vii) sufficient organ function; and (viii) written informed consent.

The exclusion criteria were as follows: (i) synchronous or metachronous malignancy; (ii) pregnant or breastfeeding women; (iii) active or chronic viral hepatitis; (iv) active bacterial or fungal infection; (v) diabetes mellitus; (vi) systemic administration of corticosteroids; and (vii) unstable hypertension.

In all cases, the pathological stage of the disease was determined as per the 7th edition of the Union for International Cancer Control TNM Cancer Staging Manual^51^.

Blood samples were obtained from the patients before surgery, and serum samples were stored. We collected 95 clinical serum samples from patients with a benign disease or with esophageal, gastric, or colorectal cancer. In this report, we used a portion of the clinical serum samples obtained from patients with benign diseases, gastric cancer, or colorectal cancer.

### Preparation of biochip

Details of the preparation of the biochip, which we designated the Proteo®chip, were described in our first report^43^. Examination plates with a round-shaped chip made of phosphor bronze (JIS H3110, C5191P) were prepared for sample analysis (Fig. 1a). Sodium thiosulfate pentahydrate (Na_2_S_2_O_3_ · 5H_2_O, Wako Pure Chemical Industries, Ltd., Tokyo, Japan) was dissolved in distilled water. Silver (I) chloride (AgCl, Wako Pure Chemical Industries, Ltd., Tokyo, Japan) was added to the solution and dissolved in a 3:1 molar ratio of Na_2_S_2_O_3_ · 5H_2_O and AgCl. The final concentration of the resulting silver thiosulfate (Ag(S_2_O_3_)_2_) was adjusted to 0.1% with distilled water. We applied 20 μL of the Ag(S_2_O_3_)_2_ solution on the chip; within a few minutes, silver NHCs were produced on the chip. The specificity of these silver NHCs has also been previously reported by Yamamoto *et al.*^52^. Although the NHCs gradually grow in size with time, no notable difference was observed in the intensity of the SERS spectrum of silver NHCs obtained at 3 and 30 min after applying the Ag(S_2_O_3_)_2_ solution. Therefore, we used NHCs obtained 3 min after applying the Ag(S_2_O_3_)_2_ solution onto the chip. Any excess Ag(S_2_O_3_)_2_ solution after 3 min was removed by blow drying. Next, sodium hypochlorite was added onto the chip, and excess sodium hypochlorite solution was removed 3 min later by blow drying. Before measurement, 10 μL of the sample was added onto the chip, and excess sample was removed 1 min later by blow drying. Finally, a negatively charged silver NHC chip was completed. Although positively charged rhodamine 6G can bind to the silver NHC chip, negatively charged Congo red cannot.^43^

### Clinical serum sample preparation

A 5.0-mL sample of peripheral vein blood was obtained from each patient before surgery. The blood sample was drawn into tubes containing a clot activator and a polyolefin gel (Venoject II, VP-AS109K50, Terumo Corporation, Tokyo, Japan), and then centrifuged at 1600×g by using a centrifuge separator (Model 5930, Kubota Corporation, Tokyo, Japan) for 7 min at room temperature. The serum extracted from each blood sample was stored at –80 °C. In our first study, we confirmed that the measurement chip became saturated with clinical serum samples diluted 10- to 100-fold.^43^ Therefore, we diluted each clinical serum sample 10- to 100-fold with distilled water prior to analysis. The components in each serum sample, including single-strand DNA, double-strand DNA, RNA, and protein, were calculated by using a Qubit2.0 fluorometer (Life Technologies Japan Ltd., Tokyo, Japan).

### Cultured cell sample preparation

Cultured cells including Kato III (RCB2088, human gastric signet ring carcinoma cell line), MKN45 (RCB1001, human poorly differentiated gastric adenocarcinoma cell line), CW-2 (RCB0778, human colon carcinoma cell line), PK-45P (RCB2141, human pancreatic carcinoma cell line), and NHDF-Neo (CC-2509, normal human dermal fibroblasts) were used in this study. Kato III, MKN45, CW-2, and PK45-P were provided by the RIKEN Bioresource Center through the National Bio-Resource Project of the MEXT, Japan. NHDF-Neo was purchased from Lonza Japan Ltd., (Tokyo, Japan). We initially prepared two samples from the cultured cells, one of which consisted of cells chemically lysed by using guanidine isothiocyanate, β-mercaptoethanol lysis buffer (Buffer RLT, Qiagen, Valencia, CA), and QIAshredder (Qiagen). The samples were finally adjusted to 10^5^ cells/ 600 μL according to the manufacturer’s protocol. The other sample consisted of cells physically fractured by passing them through a sterilized 26-gauge needle (NN-2613R, Terumo Corporation, Tokyo, Japan) ten times. The number of cells in both samples were counted by using a TC10 automated cell counter (Bio-Rad Laboratories Inc., Hercules, CA), and finally adjusted to 10^5^ cells/ 600 μL with dilution by distilled water.

### Preparation of DNA, RNA, and protein samples

By using AllPrep DNA/RNA/Protein Mini Kit (Qiagen), DNA, RNA, and protein samples were extracted from the chemically lysed culture cells (including KATO-III, MKN45, CW-2, PK-45P, and NHDF-Neo) according to the product protocol, and were calculated by using a Qubit2.0 fluorometer.

Morphological and compositional analysis of chip surface by using field emission–type scanning electron microscope

The components of the surface of the chip were analyzed by using a field emission–type SEM (JSM-7001F, JEOL Ltd., Tokyo, Japan). In addition, we used the SEM (JSM-7001F) to analyze the transition of the surface structure on the silver NHC chip, after adding drops of the sodium hypochlorite solution, and after adding 10 μL of the clinical serum sample obtained from a patient with gastric cancer, diluted 10-fold with distilled water.

Shape and RGB color histogram analysis of structure on surface of silver NHC chip by using 3D laser scanning confocal microscope.

The shape and RGB color histogram of the chip surface were recorded using a 3D laser scanning confocal microscope with a 408-nm wavelength violet laser light source and white light source (VK-X 250, Keyence Corporation, Osaka, Japan). Ten microliter samples of clinical serum diluted 10- or 100-fold; chemically lysed cultured cells; extracted DNA, RNA, or protein, and physically fractured cells were applied onto the chip. Excess sample was removed 1 min later by blow drying. All samples were initially observed in ×100 view, and the largest nodule was observed at large magnification (×3000 or ×1000) and in 3D view.

### Measurement of SERS spectrum of cultured cell samples

We measured the Raman spectra of chemically lysed cultured cell samples as well as samples of extracted DNA, RNA, protein, and physically fractured cells. Each 10-μL sample was added onto the chip, and distilled water was used as the negative control. Two types of laser Raman microscope were used to measure SERS spectra. One was equipped with automatic focus control and a computer-controlled moving stage (50× objective lens, focal length 100 mm, Andor DV420A-OE CCD camera; RAM-300, Lambda Vision, Inc., Sagamihara, Kanagawa, Japan). A helium–neon laser of 632.8-nm wavelength was used at 2 mW. For each sample, the SERS spectrum was measured once a second for each of the 100 points in a 1-mm^2^ area. The measurement area was microscopically adjusted to measure the most number of nodules, including the largest one on the chip surface. The spectrum at peak intensity was then recorded. The other Raman microscope was equipped with automatic focus control and a direct laser shooting system by surface imaging (DXR Raman microscope, Thermo Fisher Scientific Inc., Waltham MA). This microscope could irradiate any point by observing the enlarged image of the chip surface. Therefore, we used it to obtain the SERS spectrum by aiming the laser at a crystal on the chip.

## Additional Information

**How to cite this article**: Ito, H. *et al.* Silver Nanoscale Hexagonal Column Chips for Detecting Cell-free DNA and Circulating Nucleosomes in Cancer Patients. *Sci. Rep.* **5**, 10455; doi: 10.1038/srep10455 (2015).
